# Role of translational noise on current reversals of active particles on ratchet

**DOI:** 10.1038/s41598-023-42066-5

**Published:** 2023-09-27

**Authors:** Anshika Chugh, Rajaraman Ganesh

**Affiliations:** 1 Institute for Plasma Research, Bhat, Gandhinagar, 382428 India; 2 Homi Bhabha National Institute, Training School Complex, Anushaktinagar, Mumbai, 400094 India

**Keywords:** Condensed-matter physics, Statistical physics, thermodynamics and nonlinear dynamics

## Abstract

In this study, we explore using Langevin dynamics simulations, the role of thermal fluctuations on the rectification of non-interacting inertial active (self-propelled) particles in a rocking ratchet setup in the absence and in the presence of the external time periodic drive. The system is first studied in the absence of the external drive. It is found that the average velocity is always positive and a peaked function of the translational noise, indicating that the asymmetry effects dominate at intermediate values of the strength of the thermal noise. In the second part of this work, we study the effect of the external drive on the dynamics of the system by exploring a phase diagram in the parameter space of translational noise and driving frequency for two different strengths of rotational diffusion. For a given constant amplitude of the active force and amplitude of external drive less than the maximum force due to the potential, the average velocity magnitude as well as the direction ($+ {\hat{x}}/- {\hat{x}}$) is found to depend on the rotational diffusion, frequency of the external drive and the strength of the translational noise. We discover certain critical parameters in the phase space at which current reversals happen. It is found that when the average particle energy is lower than the potential energy of the barrier, symmetry breaking dominates and the currents are in the ‘easy’ direction of the ratchet. On the other hand, when the energy available per particle crosses the potential energy of the barrier, the competition between inertial effects and diffusion effects decides the direction of currents. We explain our findings by constructing phase difference datum, velocity probability distribution, and current probability analyses. Our results provide a novel method for controlling the direction of transport of inertial active particles.

## Introduction

Rectifying unbiased fluctuations into directed motion is termed as a ratchet effect^1^. The Ratchet effect was first observed experimentally by Rousselet et al.^2^, which demonstrated the transport of colloidal particles in a solution. Subsequently, ratchet effects have been reported and extensively studied both theoretically and experimentally for a variety of systems such as vortices and Josephen phase in superconductors^3–5^, electrons on semicondcutors^6,7^, colloidal matter^8^, granular matter^9^ and dusty plasma^10,11^. All these systems employ an external drive to break symmetries in the system. However, active matter ratchets^12^ offer a new class of rectification mechanism, which unlike driven or passive systems does not require external drive forces for directed motion. Active matter ratchets can be realised using biological particles such as bacteria or synthetic active particles, for example, Janus particles^13^. Active particles, unlike passive particles, have internal mechanisms to convert energy fluctuations to their own directed motion. The consequences of self-propulsion on rectifying effects have led to an emerging interest in the field of active matter ratchets.

Studies have shown how particle self-propulsion can be exploited to sort swimming bacteria in funnel arrays^14^, sort sperm cells with the use of microfluidic trapping chips^15^, transport particles over asymmetric substrates, or asymmetric obstacles^16–22^. Some studies also investigate reverse ratchet effects by utilizing the inertia of active particles^19^, leveraging particle alignment^22^ or employing time-oscillating potentials^23^. Understanding the transport properties of active entities is of great importance for various biomedical applications. An experimental study demonstrates, how curved asymmetric obstacles can be used to create highly efficient microratchets using the geometrical guidance and trapping transition of human sperm cells in quasi-2D microchambers to control their distribution^24^. Bulk of the studies on active ratchet systems explore the role of activity or self-propulsion on the transport properties of the system. However, the effect of thermal fluctuations on the system remain largely unexplored. A more realistic approach to ratchet problems involves taking into account the background fluctuations as transfer of chemical energy to electric or mechanical energy at mesoscopic scales is known to occur in the presence of random fluctuations. A recent study^25^ demonstrates that the motility of particles can be controlled using ambient thermal fluctuations. Thus, the strength of thermal fluctuations can act as an efficient control parameter to drive dynamics of the system.

In situations such as self-propelling microdiodes^26^, dusty plasma, granular matter in dilute systems, inertia can have significant effect on the dynamics of the system, for example, interplay between inertia and self-propulsion can produce smoother trajectories and modify the thermodynamical properties^27^. In inertial limit, active particles display distinctive non-linear behaviour^28,29^.

In our previous study, we had shown the emergence of directed motion for inertial interacting passive Yukawa particles on rocking ratchet. In our present study, we investigate inertial non-interacting active particles in the same ratchet set-up as our previous paper. Though the two studies consider different particles (passive/active) and different interaction limits, we want to emphasize that directed motion is possible in either scenario. The passive particles utilize external drive to observe directed motion while active particles can undergo directed motion without the need of an external drive. Therefore, the present study explores the combined effect of active force and the external drive on the directed motion of the active particles. Hence, for simplicity and to isolate the effect of interactions on the dynamics, we ignore interactions among the particles and study thermal effects on the dynamics of the active system. It is found that for active particles on a stationary ratchet, positive currents are observed across different strengths of thermal noise indicating that particles favour motion in the ‘easy’ direction of the ratchet where the magnitude of force required to cross the barrier is less as compared to the other direction. Below a critical noise parameter, thermal fluctuations help in directed motion, and above the parameter, directed motion is reduced. For active particles on rocking ratchet, we explore the phase diagram in noise parameter and rocking frequency space and observe current reversals for a certain set of parameters. It is found that when total average energy of the system impedes the potential barrier, symmetry-breaking effects dominate and current flows in the easy direction of the ratchet whereas current in the hard direction of the ratchet is due to the interplay of inertial and diffusion effects in the system. In the limit, when the strength of thermal fluctuations equals potential barrier height, we recover the passive dynamics of the system. We believe that the novelty of our work lies in the observation of the current reversals with variation in the strength of the thermal fluctuations. The study might have implications on the way the transport properties are controlled using the ambient thermal fluctuations.

In Section “Model”, we introduce the model. The results are shown and discussed in Section “Results and discussion”. Finally, the conclusions are drawn in Section “Conclusion”.

## Model

**Figure 1:**
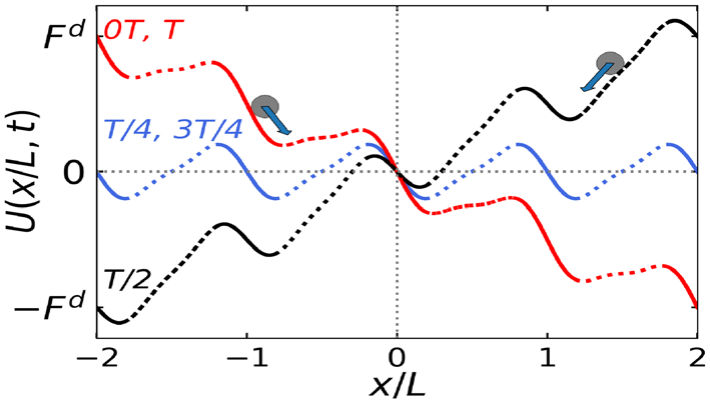
Pictorial representation of the rocking ratchet, $U(x,t) = U(x) - A_d cos(\Omega t)x)$ at different times 0 $\Im$, $\Im/4$, $\Im/2$, $3\Im/4$ and $\Im$. *U*(*x*) denotes the profile of the standing ratchet potential given by, $U(x) = -(sin(2 \pi x/L) + 0.25 sin(4 \pi x/L))$ (blue in color). The ratchet is rocked periodically with time period $\Im$ and frequency $\Omega= 2 \pi /\Im$. Dashed and solid lines respectively show the easy and hard direction of the ratchet profile.

We perform Langevin dynamics simulations on self-propelled point particles in a rectangular box of dimensional ratio $L_x/L_y = 2 / \sqrt{3}$ with periodic boundary conditions in both directions, using an in-house developed Molecular Dynamics solver MPMD^30^. In this 2D box, we apply an asymmetric potential, called ratchet, $U(x) = U(x + L)$ with period L, along the *x*-direction. We introduce 32 ratchet cycles along *x*-direction such that, $L_x = 32 L$. The ratchet is rocked periodically using a cosine drive of frequency $\Omega$ and amplitude $A_d$ (Fig. 1). In this work, we ignore the interactions among the active particles to study the single-particle behaviour on ratchet under the effect of thermal fluctuations and periodic driving. The dynamics of the particles, each of mass *m*, position $(x_i, y_i)$, and the self-propulsion direction $\theta _i$, are governed by the following active Langevin equations:

1 $$\begin{aligned} m \ddot{x_i}&= -\gamma \dot{x_i}+ \sqrt{2 \gamma k_B T}{\chi }_i (t) + f^{a} cos(\theta_i ) + f^{R}_{i} + f^{d}_{i}, \end{aligned}$$

2 $$\begin{aligned} m \ddot{y_i}&= -\gamma \dot{y_i}+ \sqrt{2 \gamma k_B T}\chi _i (t) + f^{a} sin(\theta_i ) , \end{aligned}$$

3 $$\begin{aligned} {\dot{\theta }}_i&= \sqrt{2 {\hat{T}}_r} \xi _i(t). \end{aligned}$$

$\gamma$ is friction coefficient, *T* is ambient temperature, $D_r$ is the angular diffusion coefficient, $\xi$ and $\chi _i$ are the Gaussian white noise with zero mean and unity variance. $\chi$ mimicks the Langevin kicks from fluid-like background medium. $f^{R}_{i} = \frac{\partial U(x_i)}{\partial x_i}$ denotes the force due to ratchet potential $U(x) = -(sin(2 \pi x/L) + 0.25 sin(4 \pi x/L))$, $f^{d}_i = A_d cos(\Omega t)$ is the force due to an external periodic drive. The ratchet together with the external periodic drive is called a rocking ratchet. The active particles move persistently with constant speed $\text {v}_0 = f^a / \gamma$ along the self-propulsion angle $\theta$ for an average time $\tau _p = 1/D_r$. The average time $\tau _p$ is the persistence time of the particles. We consider ratchet period *L* and $\tau _0 = \sqrt{m L^2/\Delta U}$ as the normalized unit for length and time, respectively. $\tau _0$ denotes the oscillation frequency of a particle at the bottom of the ratchet potential. Energy is measured in the unit of the potential barrier height $\Delta U = max$ $U(x) - min$ *U*(*x*). The normalized dynamical equations are:

4 $$\begin{aligned} \ddot{x_i}&= -\eta \dot{x_i}+ \sqrt{2 \eta k_BT/\Delta U}{\chi }_i (t) + F^{a} cos(\theta_i ) -\textrm{d} U(x_i)/\textrm{d}x_i + F^{d} cos(\omega t), \end{aligned}$$

5 $$\begin{aligned} \ddot{y_i}&= -\eta \dot{y_i}+ \sqrt{2 \eta k_B T/\Delta U}\chi _i (t) + F^{a} sin(\theta_i ) , \end{aligned}$$

6 $$\begin{aligned} {\dot{\theta }}_i&= \sqrt{2 D_r} \xi _i(t). \end{aligned}$$

Rescaled parameters^11^ are (a) $\eta = \gamma L/\sqrt{m \Delta U}$, reduced friction coefficient (b) $k_BT/\Delta U$, reduced temperature (c) $F^a = f^a L / \Delta U$, reduced activity strength (d) $F^d = A_d L/\Delta U$, reduced driving amplitude (e) $\omega = \Omega \sqrt{m L^2/\Delta U}$, reduced driving frequency (f) $D_r = {\hat{D}}_r \sqrt{m L^2/\Delta U}$, reduced rotational diffusion.

Note that the ratchet potential and the external periodic drive acts only along *x*-direction. Thus, to quantify the current, we measure the average velocity in *x*-direction. The averages are taken over $10^3$ ensembles and $10^5$ time periods, ${\mathscr {T}} ( = 2 \pi / \omega )$ of the external drive. We define the average velocity as:

7 $$\begin{aligned} \langle \text {v}_x \rangle&= \lim _{N_t \rightarrow \infty } \frac{\omega }{N_t 2 \pi } \int _{t}^{t + N_t\frac{2 \pi }{\omega }} \frac{1}{n} \sum _{i=1}^{n} \text {v}_{xi}(t) dt \end{aligned}$$

We integrate the equations of motion (Eqs. 4–6) using Leapfrog method with time step $\delta t$ set to $10^{-3}$. To study effectively, the role of translational noise together with an external drive, the activity strength is kept constant at $F^a = 1.5$. This ensures the absence of directed motion when both the translational noise and the external drive are neglected. In our study, we have considered underdamped regime at a fixed inertial parameter, $\gamma = 0.5$ and investigate the role of thermal fluctuations in the system. Since, the friction coefficient, $\gamma$ is constant throughout the study, increase in ambient temperature, $k_BT / \Delta U$ increases the thermal fluctuations or thermal noise, therefore we use the terms interchangeably.

## Results and discussion

The emergence of directed motion in any system involves breaking of spatial and temporal symmetries. In our system, we use an asymmetric potential called ratchet to break spatial symmetries and the source of temporal asymmetries comes from the self-propulsion of active particles or an external unbiased zero-mean force drive. Now, we will discuss two cases: (a) active particles in the absence of the external drive i.e. active particles on standing ratchet, and (b) active particles in the presence of the external drive, i.e. active particles on rocking ratchet. In the first case, active particles help break temporal symmetries and in the second case, the self-propulsion of active particles as well as the external drive help break the temporal symmetries.

### Active standing ratchet

**Figure 2:**
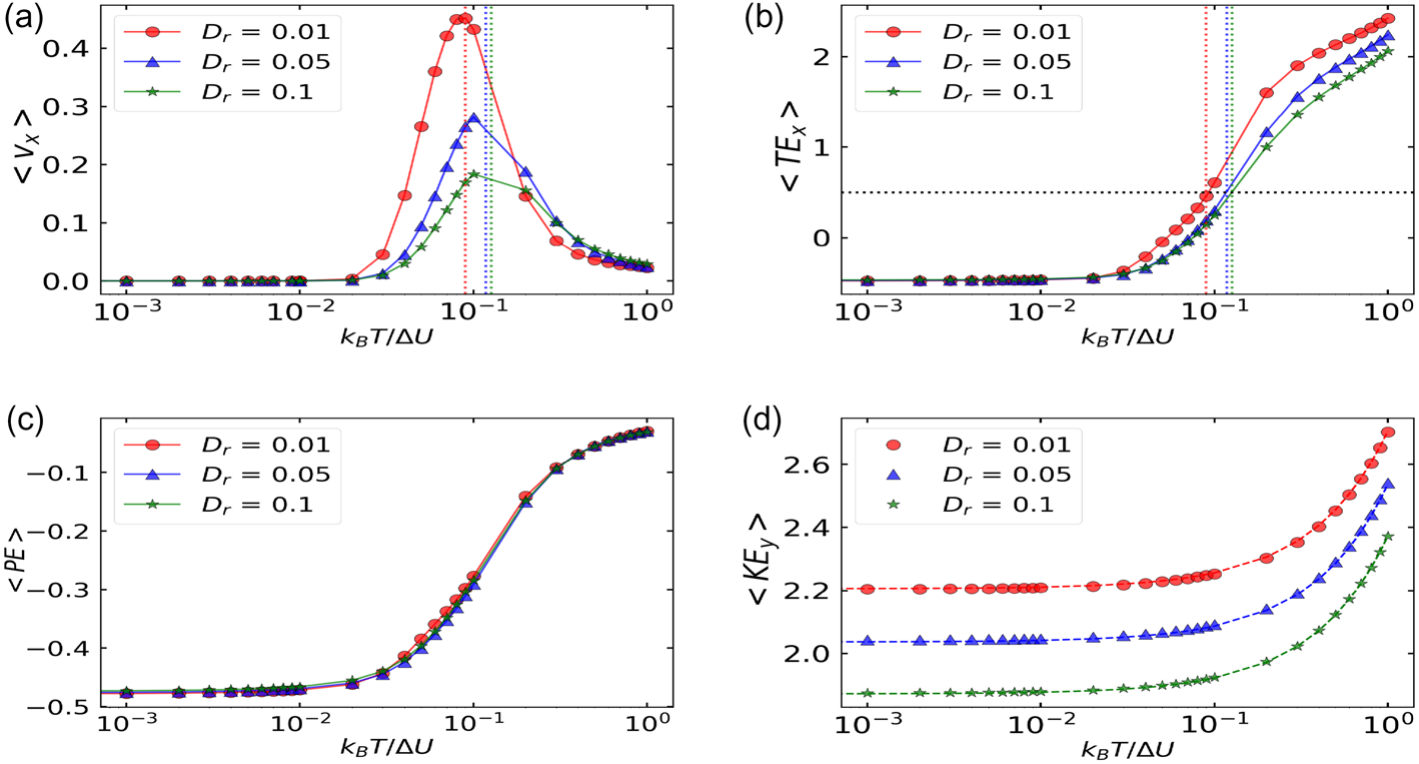
Particles’ (**a**) average velocity (see Eq. (7)), $\langle \text {v}_x \rangle$ (**b**) average total energy in *x*-direction (see Eq. (10)), (**c**) average potential energy see Eq. (8)), and (**d**) average kinetic energy in *y*-direction (see Eq. (9)), as a function of translational noise, $k_BT/\Delta U$ for different values of rotational noise, $D_r$. Dashed lines of the corresponding curves show the cross-over point of translation noise, $k_BT/\Delta U$ at which the particles average potential energy becomes equal to the maximum potential energy of the ratchet barrier (dashed black line in (**b**)). The constant parameters are: $F^a = 1.5$, $\Delta U = 1$, $\eta = 0.5$, $F^d = 0$.

Various energy quantities like average total energy, average potential energy, and average kinetic energy corresponding to *x* and *y* directions are defined as:

8 $$\begin{aligned} \langle PE \rangle&= \lim _{t_f \rightarrow \infty } \int _{t_i}^{t_f} \frac{1}{n} \sum _{i=1}^{n} U(x_i, \Im/4) dt, \end{aligned}$$

9 $$\begin{aligned} \langle {KE}_{x/y} \rangle&= \lim _{t_f \rightarrow \infty } \int _{t_i}^{t_f} \frac{1}{n} \sum _{i=1}^{n} [{(\text {v}_{xi/yi} - \langle \text {v}_{x/y} \rangle )}^2 ] dt, \end{aligned}$$

10 $$\begin{aligned} \langle {TE}_x \rangle&= \langle {KE}_x \rangle + \langle PE \rangle , \end{aligned}$$

**Figure 3:**
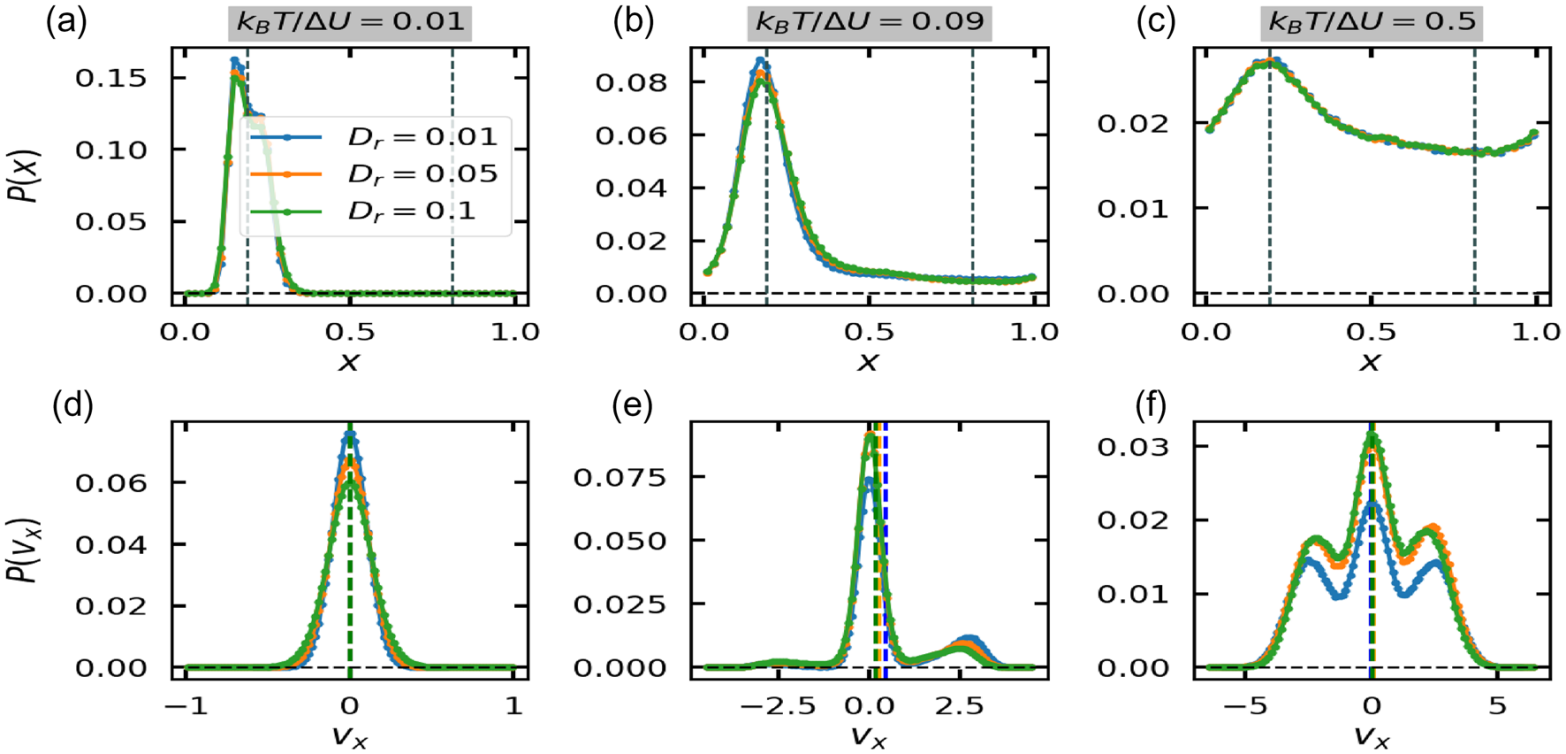
(top row) Particles’ positions distribution within one spatial period $(L = 1)$ of the ratchet potential and (bottom row) particles’ velocities distribution at three different values of noise strength ($k_BT/\Delta U = 0.01,$ 0.09, 0.5 (left to right)) and for $D_r = 0.01$(blue), $D_r = 0.05$ (orange) and $D_r = 0.1$ (green). Dashed grey lines is top row shows the position of the potential well ($x = 0.19$) and potential barrier ($x = 0.81$) respectively. Dashed lines of the bottom row in their respective $D_r$ colors shows the value of the average velocity.

In Fig. 2a, we observe that there are no currents or average velocity below $k_BT/\Delta U = 0.01$ as particles are pinned at the bottom of the potential well as shown in Fig. 2b,c), where the average potential and the total energy of the system is around the minimum potential energy of the barrier. After this point, the average velocity increases with an increase in translational noise $k_BT/\Delta U$ and the corresponding values of average total energy in *x*-direction also increases (Fig. 2b) for different values of rotational noise considered. Vertical dashed lines for different $D_r$ in Fig. 2a,b, corresponds to the values of translational noise $k_BT/\Delta U$ at which the average total energy of the particles becomes equal to the potential energy required to cross the ratchet barrier. We call these points as the cross-over points.

We note that the peak in average velocity is observed at the same location in $k_BT/\Delta U$, where the cross-point occurs. Although the average total energy in *x*-direction for the values of $k_BT/\Delta U$ less than the cross-over point, is below the maximum potential energy of the barrier, we still observe positive average velocity, i.e currents in the easy direction (right) of the ratchet. This can be explained as follows: For the considered values of $D_r = (0.01/0.05/0.1)$, the problem addressed falls into large persistence regime where the persistence time is more than the relaxation time of the velocities^31,32^, i.e. $1/D_r \gg 1/\eta$, where $\eta$ is reduced friction coefficient. Consequently, asymmetry effects dominate, that is, the probability of particles to move towards easy direction (right) is more than the probability to move towards hard direction (left) of the ratchet. In large persistence regime, the barrier crossing not only depends on the potential height but also on the force felt by the particle at the well bottom^33,34^. For the considered asymmetric potential, the magnitude of force required to cross the barrier to the right is $|-2.14|$ (i.e.$f^{M}_r$) and to cross the barrier to the left is 4.28 (i.e. $f^M_l$). The magnitude of active force ($F^a$) in the problem is 1.5 which is less than $f^M_r$ and $f^M_l$. As a result, the rare events of jumps over the barrier due to active force is assisted by thermal noise. After the cross-over point, particles gain enough energy to cross the ratchet barrier and the asymmetry effects weakens. Now, the possibility of the particles moving towards left and right becomes equally probable. Hence, the ratchet effect diminishes after the cross-over point.

Figure 3 shows that the particles are mainly distributed around the bottom of the potential well at $x = 0.19$. However, this distribution spreads out (Fig. 3b,c) with increase in the strength of the thermal noise and the corresponding velocity distribution show double and triple peak behaviour (Fig. 3e,f). At low values of noise strength, particles are unable to cross the potential barrier as shown by the absence of any particles in the region [0.35 : 1.0] (Fig.3a) and the corresponding velocity distribution shows a peak around 0 (Fig. 3d). At intermediate values of the strength of the thermal noise, particles can cross the barrier to the right as shown by the presence of a short peak in the velocity distribution curve (Fig. 3e) and positions’ distribution show a wider curve around the peak alongside the non-zero probability of finding particle in the region outside the potential barrier (Fig. 3b). Therefore, in this range of thermal noise, asymmetry effects dominate and currents are observed in the ‘easy’ direction where the amount of force required to cross the barrier is less than the other side. At large strength of thermal noise, thermal effects dominate, conversely, particles do not experience the ratchet.

As there is no ratchet mechanism along *y*-direction, the average kinetic energy in *y*-direction is equal to that of the active particles with effective temperature $T_{eff} = T + (F^a)^2 \tau _p/2 \gamma k_B$. Dashed lines for the corresponding $D_r$ in Fig. 2d are the theorectical fit of the kinetic energy of active particles with effective temperature, $T_{eff}$. We observe that the numerical data points fit well onto the theoretical curve, which confirms the correctness of our numerical calculations.

As the active force reorients faster for large $D_r$, the particles cannot cross the barrier efficiently which also explains the reduced magnitude of average velocity with increase in $D_r$. On the contrary, when $D_r$ is small, the particles can move persistently in one direction for longer times, therefore contributing to efficient crossing of potential barrier and larger energies as shown by the vertical shift of the curve in Fig. 2b.

Thus, below the cross-over points, the asymmetry effect dominates and thermal fluctuations help in rectification by providing sufficient energy to the particles. On the other hand, after the cross-over points, thermal fluctuations suppress the currents. In the next sub-section, we investigate the effect of external unbiased drive on the currents in the system.

### Active rocking ratchet

**Figure 4:**
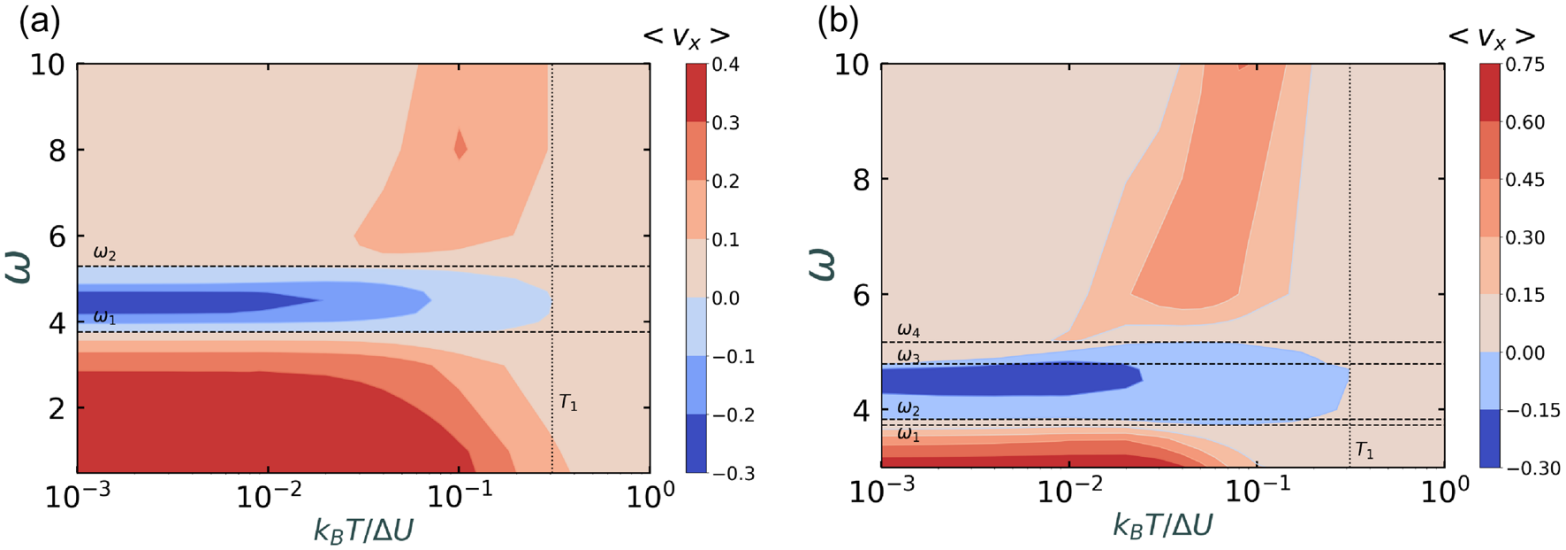
Contour plots of average velocity as a function of translation noise $k_BT/\Delta U$ and driving frequency $\omega$ for (**a**) $D_r = 0.1$ and (**b**) $D_r = 0.01$. The other parameters are $F^a = 1.5$, $F^d = 1.0$, $\eta = 0.5$, $\Delta U = 1$. The dashed and the dotted lines show the critical parameters at which the current reversal happens. The values of these critical parameters are obtained numerically. These values are $\omega _1 \approx 3.77, \omega _2 \approx 5.3, T_1 = 0.3$ for $D_r = 0.1$ and $\omega _1 \approx 3.74, \omega _2 \approx 3.8, \omega _3 \approx 4.85, \omega _4 \approx 5.17, T_1 \approx 0.31$ for $D_r = 0.01$.

**Figure 5:**
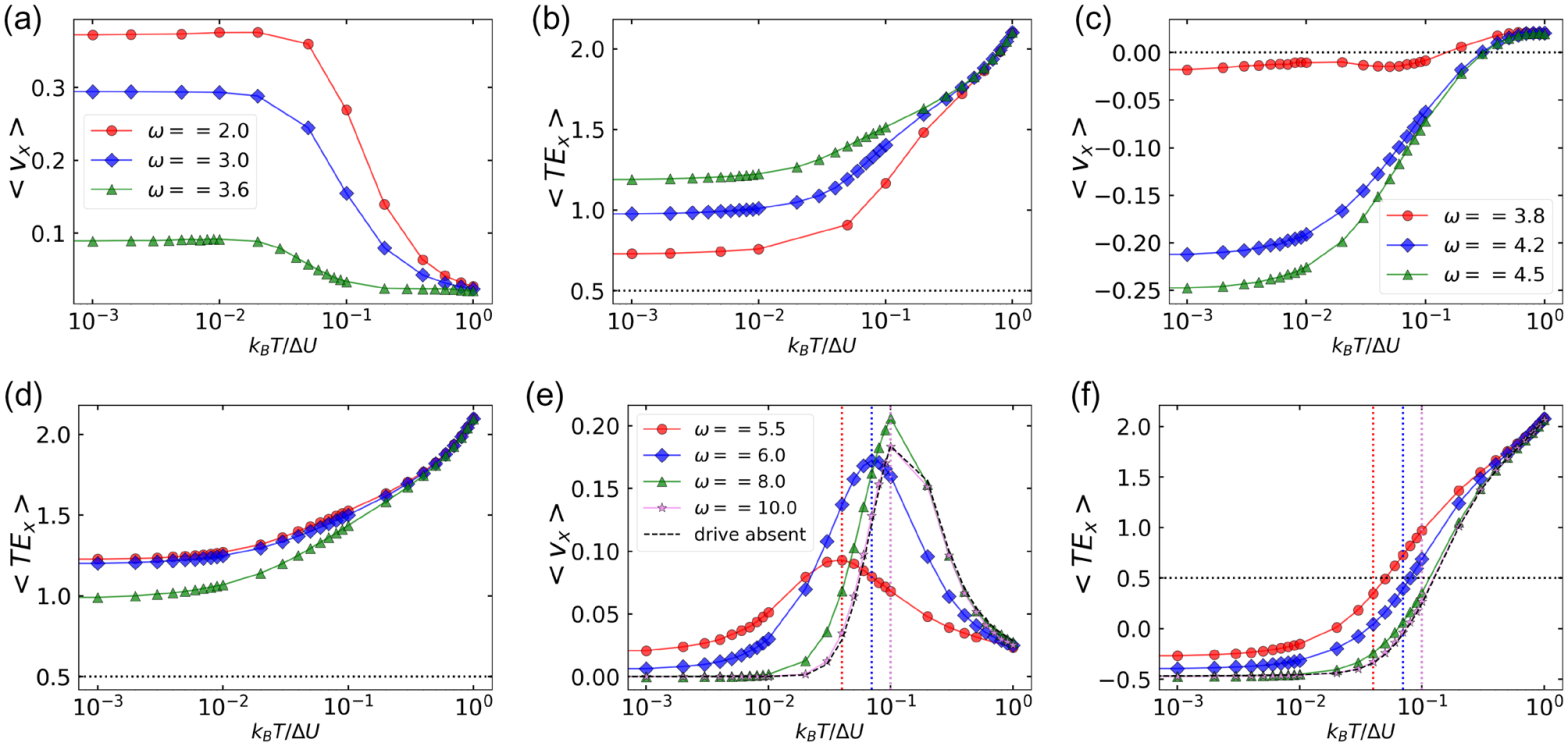
Particles’ average velocity and the corresponding average total energy in *x*-direction as a function of translational noise $k_BT / \Delta U$. (**a, b**) $\omega < \omega _1$, (**c, d**) $\omega _1 < \omega _2$ and (**e, f**) $\omega > \omega _2$. The other parameters are: $F^a = 1.5$, $\Delta U = 1$, $\eta = 0.5$, $F^d = 1.0$ and $D_r = 0.1$.

**Figure 6:**
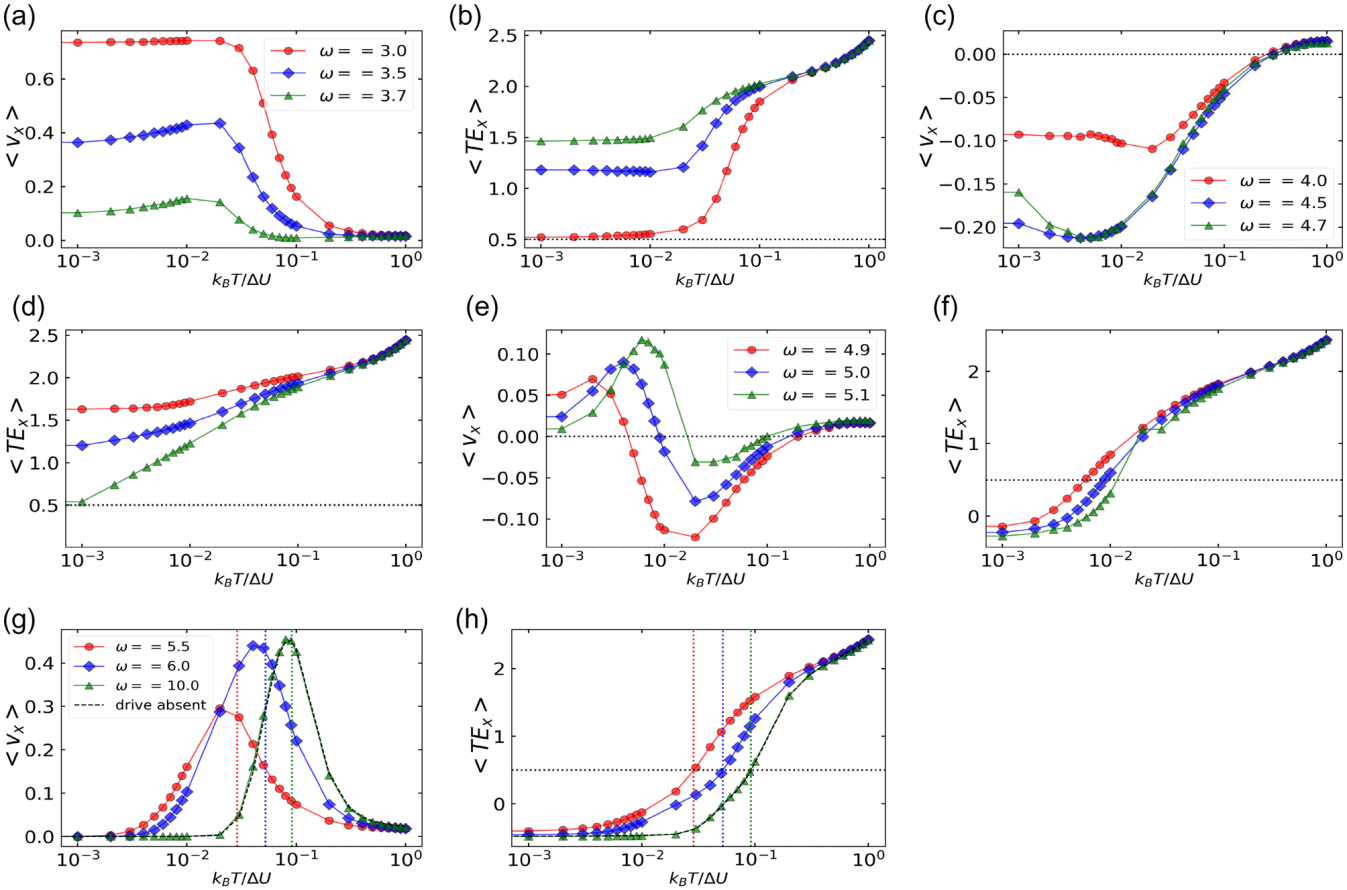
Particles average velocity and the corresponding average total energy in *x*-direction as a function of translational noise $k_BT / \Delta U$. (**a, b**) $\omega < \omega _1$ (**c, d**) $\omega _2< \omega < \omega _3$ (**e, f**) $\omega _3< \omega < \omega _4$ (**g, h**) $\omega > \omega _4$. Double reversal with change in $k_BT/\Delta U$ is observed for $\omega$ in the range $\omega _1< \omega < \omega _2$ and $\omega _3< \omega < \omega _4$. The other parameters are: $F^a = 1.5$, $\Delta U = 1$, $\eta = 0.5$, $F^d = 1.0$ and $D_r = 0.01$.

**Figure 7:**
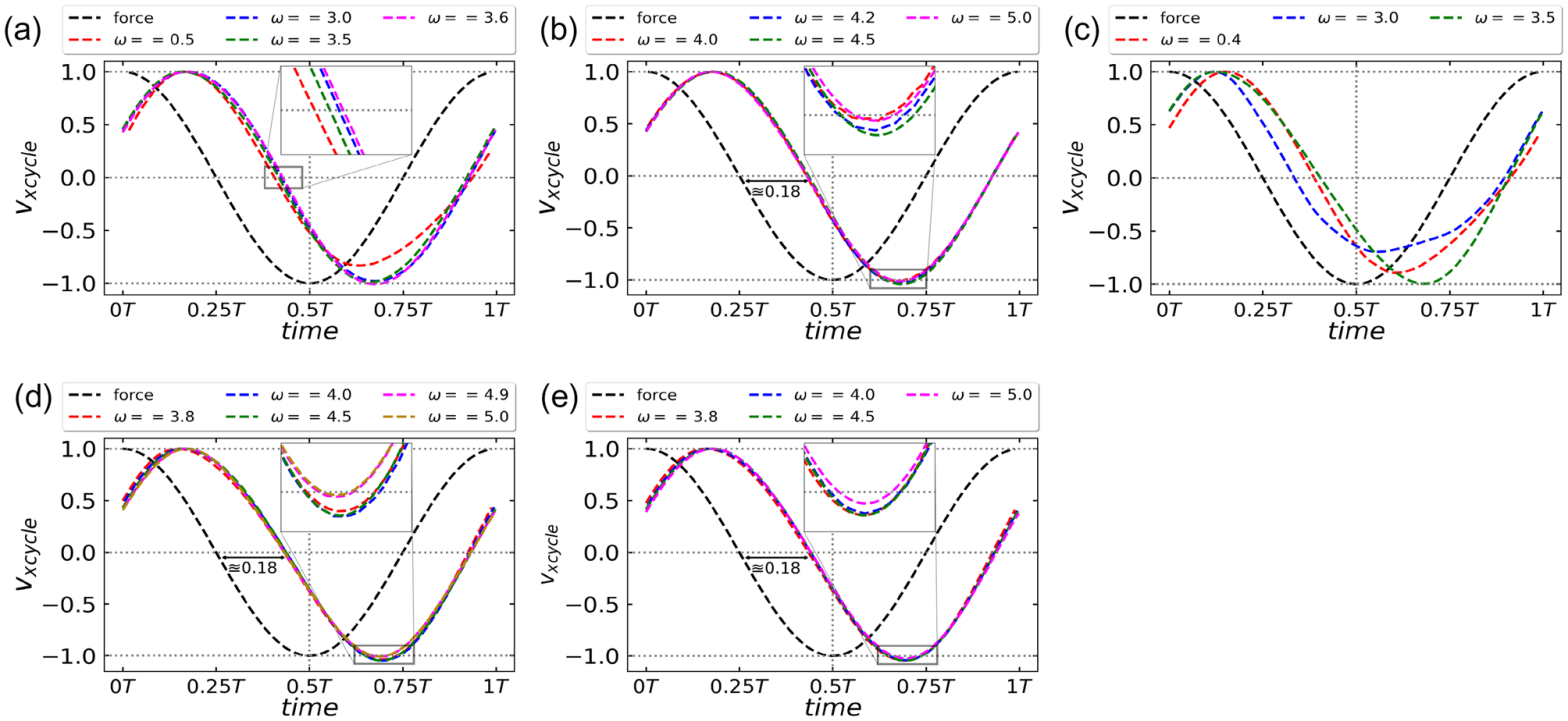
Normalised instantaneous velocity per particle, ${v_x}_{cycle}$ variation over one $k_BT/\Delta U$, *T* of the external drive, for (**a**) $\omega < \omega _1$, (**b**) $\omega _1< \omega < \omega _2$ and $D_r = 0.1$, $T = 0.005$, (**c**) $\omega < \omega _1$, (**d**) $\omega _2< \omega < \omega _3$, and $Dr = 0.01, T = 0.005$ (**e**) $\omega _3< \omega < \omega _4$ and $D_r = 0.01, T = 0.02$. Black dashed curve in each plot shows the instantaneous external force during one period of the external drive. Inset shows the zoomed plot of the indicated grey rectangular box.

**Figure 8:**
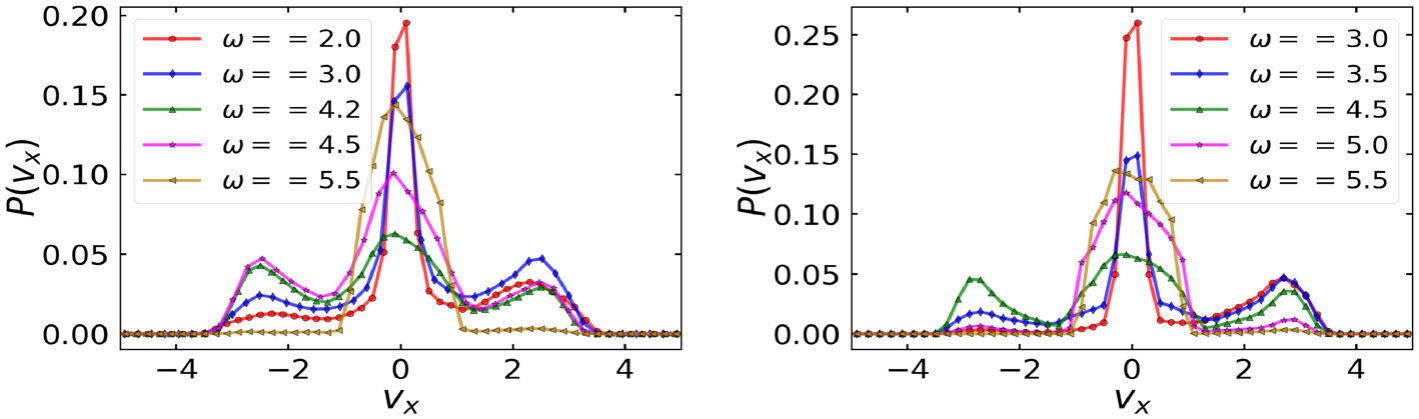
*x*-velocity distribution, $P(v_x)$ of particles for different driving frequencies, $\omega$ at $D_r = 0.1$ (left) and $D_r = 0.01$ (right) and fixed translational noise, $k_BT/\Delta U = 0.005$. Other parameters are: $F^a = 1.5$, $F^d = 1.0$, $\eta = 0.5$, and $\Delta U = 1$.

**Figure 9:**
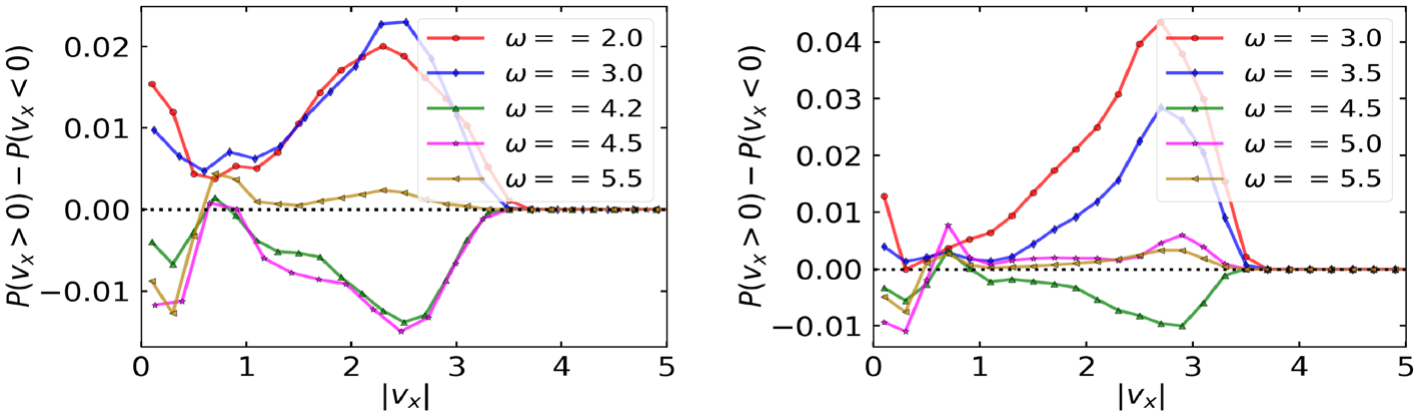
*x*-current probability defined as $|v_x|(P(v_x >0) - P(v_x < 0))$ for each value of $|v_x|$ for different driving frequencies, $\omega$ at $D_r = 0.1$ (left) and $D_r = 0.01$ (right) and fixed translational noise, $k_BT/\Delta U = 0.005$. Other parameters are: $F^a = 1.5$, $F^d = 1.0$, $\eta = 0.5$, and $\Delta U = 1$.

To study the role of translational noise, $k_BT/\Delta U$ in the presence of the external unbiased drive, we construct contour plots of the average velocity (Fig. 4) as a function of $k_BT/\Delta U$ and $\omega$ for $F^a = 1.5$, $F^d = 1.0$ and for two different orders of rotational diffusion, $D_r = 0.1$ and 0.01.

We define several critical parameters in $k_BT/\Delta U - \omega$ space, marked as $\omega _1$, $\omega _2$, $\omega _3$, $\omega _4$ (dashed horizontal lines) and $T_1$ (dotted vertical line) in Fig. 4 for $D_r = 0.1$ and 0.01. The values of these critical parameters are obtained numerically. These values are $\omega _1 \approx 3.77, \omega _2 \approx 5.3, T_1 = 0.3$ for $D_r = 0.1$ and $\omega _1 \approx 3.74, \omega _2 \approx 3.8, \omega _3 \approx 4.85, \omega _4 \approx 5.17, T_1 \approx 0.31$ for $D_r = 0.01$. At these critical points, current reversals happen, that is average velocity changes its direction from positive to negative or vice-versa. Positive currents are shown in different shades of red while the negative currents are shown in different shades of blue.

For $D_r = 0.1$ (Fig. 4a), when $k_BT/\Delta U$ is increased from 0.001 till 1, average velocity is always positive for $\omega < \omega _1$ and $\omega > \omega _2$. The average velocity changes its direction once from negative to positive for $\omega _1< \omega < \omega _2$. On the other hand, when $\omega$ is increased from $\omega = 0.1$ till $\omega = 10$, the average velocity changes its direction twice (positive $\rightarrow$ negative $\rightarrow$ positive) for $k_BT/\Delta U < T_1$, while for $k_BT/\Delta U > T_1$, average velocity is always positive. Our findings show that the transport behaviours are different for the cases (*a*) $\omega > \omega _1$, (*b*) $\omega _1< \omega < \omega _2$ and (*c*) $\omega > \omega _2$. We obtain different natures of the velocity curves in each case as shown in Fig. 5a,c,e. When $\omega > \omega _2$ (Fig. 5e), the velocity curves are same in nature as that for the standing ratchet case discussed in Section (Active standing ratchet). In the limit of large $\omega$($\omega = 10$), the ratchet oscillates very fast such that the particles are not able to perceive the variation in $\omega$ and therefore the particles see a seemingly stationary ratchet and the effect of the external drive vanishes. Thus, in the limit of large $\omega$ ($\omega = 10$), the velocity curve overlaps with that of the active standing ratchet (black dashed curve in Fig. 5e). This also explains the right shift in the peak of the velocity curve when $\omega$ is increased. As also explained in Section “Active standing ratchet”, we again establish the match between the cross-over points and the peak points at which the average velocity attains its maximum for different $\omega > \omega _2$ (Fig. 5f).

For $\omega < \omega _1$ ($\omega = 2.0,3.0$ and 3.6) in Fig. 5a, we observe positive currents while for $\omega _1< \omega < \omega _2$ ($\omega = 3.8, 4.2, 4.5$) in Fig. 5c, we observe negative currents. The corresponding average total energies in *x*-direction for both the cases (Fig. 5b,d) are always greater than the ratchet potential barrier for all values of $k_BT/\Delta U$. The opposite velocities in the two cases can be explained based on the competition between the inertial effects and the dynamics due to diffusion. Due to inertia, a phase difference is introduced between the velocity per particle and the driving force, during the period of external driving, that is the velocity per particle does not faithfully follow the driving force and lags by a phase difference dictated by the value of $\omega$ and $D_r$ (Fig. 7). It is found that the inertial effects favor easy (right) direction of motion (Fig. 7a,c). On the other hand, diffusion dynamics favors hard direction of motion as particles need to travel less distance in the hard (left) direction of the ratchet than in the easy (right) direction.

For $D_r = 0.01$ (Fig. 4b), when $k_BT/\Delta U$ is increased from 0.001 till 1, average velocity is always positive for $\omega < \omega _1$ and $\omega > \omega _4$. The average velocity changes its direction once from negative to positive for $\omega _2< \omega < \omega _3$ and twice (positive $\rightarrow$ negative $\rightarrow$ posititve for $\omega _1< \omega < \omega _2$ and $\omega _3< \omega < \omega _4$. On the other hand, when $\omega$ is increased from $\omega = 3.0$ till $\omega = 10$, we observe multiple reversals for $k_BT/\Delta U < T_1$, while for $k_BT/\Delta U > T_1$, average velocity is always positive.

Similar to the case of $D_r = 0.1$, the transport behaviours are different in different regimes of the critical parameters. For $\omega > \omega _4$, the ratchet oscillates fast enough to appear as stationary. Thus, the nature of velocity curve is similar to that of the standing ratchet (Fig. 6g) and the cross-over points (Fig. 6h) also match with peak velocity points (Fig. 6g). In the limit of $\omega = 10$, there is a overlap of the velocity curve with the standing ratchet result (black dashed line in Fig. 6g) which also confirms the correctness of our results. For $\omega < \omega _1$ (Fig. 6a), inertial effects dominates which favors the current in the easy direction. For $\omega _2< \omega < \omega _3$ (Fig. 6c), diffusion dominates the dynamics and favors the current in the hard direction of the ratchet. For $\omega _3< \omega < \omega _4$ (Fig. 6e), symmetry breaking dominates for values of $k_BT/\Delta U$ where the average total energy in *x*-direction is lower than the potential barrier energy (Fig. 6f) and for larger values of $k_BT/\Delta U$, diffusion dynamics dominates. Therefore, currents are positive for values of $k_BT/\Delta U$ less than the cross-over point and negative otherwise.

To summarise, it is a combination of symmetry breaking, inertial and diffusion effects that decides the dynamics of the systems for different range of driving frequency$\omega$, and translational noise, $k_BT/\Delta U$. We will elaborate on the possible explanation for the behaviour of the velocity curves in the next Section.

### Inertial vs diffusion effects

In the $k_BT/\Delta U - \omega$ phase space, for $\omega > \omega _2$ and $\omega > \omega _4$ for $D_r = 0.1$ and 0.01 respectively (Fig. 4), the symmetry breaking due to the ratchet dominates. The behaviour of velocity curve in this case has already been discussed in the previous Sections "Active standing ratchet" and "Active rocking ratchet". In this section, we will discuss about the specific regions in $k_BT / \Delta U - \omega$ phase space where the average total energy in *x*-direction is greater than the potential energy of the barrier (Fig. 6b,d,f) such that the asymmetry effects due to the ratchet can be neglected. These specific regions are $\omega < \omega _2$ for $D_r = 0.1$ and $\omega < \omega _4$ for $D_r = 0.01$. The observation of positive and negative currents in these regions can be attributed to the competition between inertial and diffusion effects. Due to inertia, a phase lag is introduced between the velocity per particle and the external driving force. To compare the shape of these velocity curves for different values of $\omega$, we plot velocity per particle with respect to the maximum value of the velocity per particle during one cycle of external drive. We call this velocity as ${v_x}_{cycle}$. For a constant $k_BT/\Delta U < T_1$, it is found that, for values of $\omega$ less than the critical parameter $\omega _1$, the phase lag between ${v_x}_{cycle}$ and external driving force is a function of $\omega$ (Fig. 7a,c) for both $D_r = 0.1$ and 0.01. With decrease in $\omega$, the ratchet oscillates slowly which allows particles’ velocities to relax during each stage of the ratchet oscillation. Thus, when the particles face the hard direction of the ratchet in the interval [$\Im/2$, $3\Im/4$] (Fig. 1), particles with insufficient energy to cross the potential barrier get pinned. The corresponding average total energy in *x*-direction is lower for smaller $\omega$ (Figs. 5b, 6b), therefore average velocities increase with decrease in $\omega$ for a constant $k_BT/\Delta U < T_1$. On the other hand, for a constant $\omega$, with the increase in $k_BT/\Delta U$, $\langle v_x \rangle$ decreases as thermal fluctuations become dominant and suppress the contribution from the driving force.

In the parameter space $\omega _1< \omega < \omega _2$ for $D_r = 0.1$ and $\omega _2< \omega < \omega _4$ for $D_r = 0.01$, we observe a constant phase lag of 0.18 between the velocity and the force curve (Fig. 7b,d,e). Remarkably, this phase lag remains unaffected by the driving frequency $\omega$ for $D_r = 0.1$ and $D_r = 0.01$. This observation suggests that the diffusion effects come into play in these regions, overshadowing the influence of inertia in the system. As the distance between the potential well and potential maxima on the left is less than the distance between the potential well and the potential maxima to the right, thus due to diffusion, particles can move to the left direction is less time as compared to their motion in the right direction. As a result, average velocity becomes negative. With an increase in $\omega$, the time period of oscillation decreases and therefore particles need higher energies to diffuse faster. When the two time scales are suitable, we observe peaks corresponding to the maximal value of negative currents (Fig. 6b). The signature of negative currents can also be seen in the inset of plots in Fig. 7b,d,e. The inset shows that the normalised velocity per particle, ${v_x}_{cycle}$ has crossed the lower bound of $-1$ indicating the presence of residual negative current.

In the region $\omega _3< \omega < \omega _4$ for $D_r = 0.01$, we observe both positive and negative currents for a range of $k_BT / \Delta U$ (Fig. 6e). The corresponding total energies in *x*-direction (Fig. 6f) show that the cross-over points approximately match with the velocity reversal points. This indicates that below the cross-over points where the average total energy in *x*-direction is less than the potential energy of the barrier, symmetry breaking of the ratchet dominates and we observe currents in the positive direction. After the cross-over points, diffusion effects dominates and negative currents are observed. In summary, our study shows the dominance of inertial effects in specific frequency ranges, while in the other ranges diffusion effects prevail. Although the phase lag due to inertia is present, its independence on the driving frequency support the significance of diffusion effects in those particular regions.

For $k_BT/\Delta U > T_1$, thermal fluctuations become dominant and suppress the contribution from the driving force. In this limit, all velocity curves roughly overlap for different values of $\omega$ and for both $D_r = 0.1$ and 0.01.

### Velocity distributions and probability currents

In this section, we interpret positive and negative currents through the velocity distribution of particles and the current probability defined as $(P(v_x >0) - P(v_x < 0))$^35^ for each value of $|v_x|$, where $P(v_x)$ represents the probability of particle having velocity $v_x$. Since the ratchet acts only along *x*-direction, we only look at the velocities distribution in *x*-direction (Fig. 8). At $\omega = 5.5$, which lies in the region $\omega > \omega _2$ for $D_r = 0.1$ and in the region $\omega > \omega _4$ for $D_r = 0.01$ in $k_BT/\Delta U-\omega$ phase space and where the symmetry breaking due to the ratchet dominates, a single prominent peak around $v_x = 0$ is observed. This peak is slightly skewed towards the right indicating positive currents. This is also reflected in the corresponding current probability curves (Fig. 9) as the net velocities are mainly positive for $\omega = 5.5$. At values of $\omega < \omega _2$ for $D_r = 0.1$ and $\omega < \omega _4$ for $D_r = 0.01$, where the inertial or diffusion effects dominate, the *x*-velocity distribution is three-peaked, with a prominent peak around $v_x = 0$, and the other short peaks at the positive and negative extremes. For positive currents at $\omega = 2.0, 3.0$ for $D_r = 0.1$ and at $\omega = 3.0, 3.5, 5.0$ for $D_r = 0.01$, the height of the right short peak is slightly greater than the left short peak. On the other hand, for negative currents at $\omega = 4.2, 4.5$ for $D_r = 0.1$ and at $\omega = 4.5$ for $D_r = 0.01$, the height of the left short peak is slightly greater than the right short peak. The same is also reflected in current probability curves of Fig. 9, where the net current probabilities are positive for positive currents and vice-versa.

## Conclusion

In this study, we have numerically investigated the ratchet effects for non-interacting inertial active particles on rocking ratchet as a function of translational noise both in the presence and the absence of an unbiased external drive. It is found that in the absence of the external drive, currents are always positive and is a peaked function of translational noise. In the presence of the external drive, transport behaviour is determined by the magnitude of the translational noise and the driving frequency. We obtain contour plots in $k_BT/\Delta U - \omega$ space for $D_r = 0.1$ and $D_r = 0.01$, dividing the phase space into distinct regions based on the critical parameters of the system. We find that the transport behaviour falls into three main categories:(*a*) When the average total energy in *x*-direction is less than the potential energy of the barrier, the asymmetry breaking due to the ratchet dominates and positive currents are observed. (*b*) When the average total energy in *x*-direction is greater than the potential energy of the barrier, at small $\omega$, the dynamics are dominated by the inertial effects in the system which introduces phase lag between velocity per particle and the external force resulting in positive currents. (*c*) When the average total energy in *x*-direction is greater than the potential energy of the barrier, at large $\omega$, diffusion effects dominate the dynamics and the negative currents are observed due to the less distance towards the hard direction. Thus, when total energies in the system are sufficient to cross the potential barrier, then it is a competition between the inertial and diffusion effects that decides the direction of the currents. Our results are supported using velocity distributions and current probability analyses. Our study provides a unique way to control direction and magnitude of the active particles’ current through ambient fluctuations. This might have implications on understanding molecular motors, cell motion, designing artificial swimmers and targeted drug delivery. In our future work, we intend to explore a phase diagram in $\omega - D_r$ parameter space, to fully comprehend how the interplay of the two competing time scales i.e. persistence time and time period of the potential affects the dynamics of the system. We also plan to investigate the effect of interactions between particles on the system dynamics. Whether interaction between active particles assists or suppresses directed motion will be an intriguing question to explore.

## Data Availability

Data is available upon request to the corresponding author.
